# Optimizing ISO standard microbiological techniques for isolating *Campylobacter* from poultry samples amidst challenges from extended spectrum beta lactamase producing *Escherichia coli*

**DOI:** 10.1371/journal.pone.0327963

**Published:** 2025-07-31

**Authors:** Keya Ghosh, Tahia Ahmed Logno, Tridip Das, Pangkaj Kumar Dhar, Damer Blake, Guillaume Fournie, Fiona Tomley, Richard A. Stabler, Burhan Lehri, Paritosh Kumar Biswas

**Affiliations:** 1 Department of Microbiology and Veterinary Public Health, Chattogram Veterinary and Animal Sciences University, Chittagong, Bangladesh; 2 School of Agricultural, Environmental and Veterinary Sciences, Faculty of Sciences and Health, Charles Sturt University, Wagga Wagga, New South Wales, Australia; 3 UKRI GCRF One Health Poultry Hub, Chattogram Veterinary and Animal Sciences University, Chattogram, Bangladesh; 4 Department of Pathobiology and Population Sciences, Royal Veterinary College, London, United Kingdom; 5 INRAE, VetAgro Sup, UMR EPIA, Universite´ de Lyon, Marcy l’Etoile, France; 6 INRAE, VetAgro Sup, UMR EPIA, Universite´ Clermont Auvergne, Saint Genes Champanelle, France; 7 London School of Hygiene and Tropical Medicine, London, United Kingdom; Tribhuvan University, NEPAL

## Abstract

Isolation of zoonotic *Campylobacter* species has been standardized through the ISO 10272:2017 protocol. However, application of the protocol in a LMIC country failed to isolate *Campylobacter* due to extended-spectrum beta-lactamase (ESBL) producing *Escherichia coli* overgrowth during the *Campylobacter* selective enrichment phase. The aim of the study was to identify the contaminants and explore ways to mitigate them. A set of 25 non-*Campylobacter* contaminants isolated from chicken cecal samples grown on modified charcoal-cefoperazone-deoxycholate agar (mCCDA) during *Campylobacter* isolation were included. All isolates were screened for species identification and the presence of selected ESBL producing genes. Minimum inhibitory concentrations of tazobactam were measured using a microbroth dilution technique. The *Campylobacter* isolation protocol was then modified to inhibit the contaminants by adding the required tazobactam supplement to Preston broth or to mCCDA. All contaminants were found to be *E. coli* carrying at least one of the ESBL-producing genes *bla*_*TEM*_*, bla*_*CTX*_ or *bla*_*SHV*_. The MIC of tazobactam sodium for ESBL-producing *E. coli* strains grown in Preston broth was at least 128 mg/L. Preston broth supplemented with tazobactam at 128 mg/L inhibited the growth of ESBL-producing *E. coli* but did not inhibit the growth of *C. jejuni* or *C. coli*. Interestingly, mCCDA plates supplemented with tazobactam at a much lower concentration of 4 mg/L could also prevent growth of ESBL-producing *E. coli* even without broth enrichment, increasing the efficiency of isolation of *Campylobacter*. Direct inoculation of cecal materials to mCCDA supplemented with tazobactam at 4 mg/L was recommended as the most cost-effective way to conduct *Campylobacter* surveillance targeting the cecal matrix instead of directly following ISO 10272:2017 protocol.

## Introduction

*Campylobacter* are gram-negative, microaerophilic, non-spore-forming bacteria that resemble a spiral, curved, or rod shape [1]. Poultry, major reservoirs of *Campylobacter*, play a key role in the high global occurrence of human campylobacteriosis. Preliminary contamination of broiler (chickens reared for meat) flocks occurs through horizontal transmission where the organism commonly acts as a commensal, probably supported by their thermotolerant features [2]. *Campylobacter* colonization of poultry can be determined by isolation from feces. Detection of *Campylobacter* carriage before slaughter can help limit the pathogen’s spread to the human food chain. However, isolation of *Campylobacter* is challenging compared to many other pathogens linked to food-borne illness due to its fragile and complex nature, microaerophilic atmospheric requirements, sluggish growth rate, low bacterial numbers, and fastidious growth requirements [1,3].

The International Organization for Standardization protocol for Microbiology of the food chain horizontal method for detection and enumeration of *Campylobacter* spp. [4] is a standard optimized protocol used globally to isolate *Campylobacter* from products intended for human consumption. The protocol can also be applied to feeds intended for animals, and to environmental samples from areas of food production and food handling. The protocol uses Bolton or Preston broths to enrich *Campylobacter*, followed by modified charcoal cefoperazone deoxycholate agar (mCCDA) as a second selective medium [4,5]. Cefoperazone, a beta-lactam antibiotic, is used to prevent the growth of competing flora since *Campylobacter* is intrinsically resistant. Common selective agents also used in conventional *Campylobacter* agars include Cefoperazone, Cycloheximide, Trimethoprim, Rifampicin, Vancomycin and Polymyxin B [6] Possible contaminating or co-existing organisms in the sample matrix can only grow when they can hydrolase these antibiotic or resist it by any other mechanism(s). Recently, detection and selective culture of *Campylobacter* has become more difficult due to the high occurrence of antimicrobial resistant bacteria, specifically extended spectrum β-lactam resistant *E. coli*, which has been identified as a viable contaminant in studies using mCCDA and enrichment broths [7–9]. When ESBL producers are a problem, the use of suitable beta lactamase inhibitors like Tazobactum, Polymixin B, Potassium clavulanic acid and Triclosan, is recommended [7,9,10–13]. However, among all beta lactamase inhibitors, Tazobactum was found to be most effective to inhibit ESBL activity due to it’s chemical stability and cheaper cost [13].

This study aimed to characterize contaminating organisms that can grow in Preston broth or on mCCDA during surveillance for *Campylobacter* from chicken cecal contents following the ISO 10272:2017 protocol and to modify the protocol for efficient isolation of Campylobacter by inhibiting the growth of these challenging organisms.

## Materials and methods

### Samples

This study was part of the UKRI Global Challenges Research Fund (GCRF) One Heath Poultry Hub, an impact-driven development research programme working in Bangladesh, India, Sri Lanka and Vietnam (www.onehealthpoultry.org/). As part of surveillance for the zoonotic pathogen *Campylobacter* spp. within poultry, 25 non-*Campylobacter* isolates recovered from mCCDA after initial enrichment of chicken cecal contents in Preston broth were selected for study.

### Confirmation of contaminants

Non-*Campylobacter* isolates grown on mCCDA were initially streaked onto MacConkey agar and incubated at 37°C for 24 hours aerobically. After the incubation, large pink colonies were present. Suspected *E. coli* isolates were inoculated onto EMB agar and incubated at 37°C for 24 hours aerobically. The presence of colonies defined by a metallic sheen supported identification as *E. coli*, confirmed subsequently by PCR to detect the housekeeping gene *adk* (adenylate kinase) [14].

### Screening of ESBL producing genes

All the 25 isolates were tested for the presence of genes responsible for producing *blaTEM*, *blaSHV*, *blaCTX* ESBL separately with uniplex PCR, using the primers as shown in (Table 1).

**Table 1:** Primers used in PCR to detect *hipO*, *glyA* and 23S rRNA gene sequences as markers for confirmation of *C. jejuni*, *C. coli* and *Campylobacter* spp. Respectively, and for the presence of *adk, bla*_*TEM*_, *bla*_*SHV*_, *bla*_*CTX*_ genes in *E. coli.*

Gene	Primer name	Primer sequence (5΄-------- 3΄)	Amplicon size(bp)	Annealing temp	References
*adk*	AdkF	ATTCTGCTTGGCGCTCCGGG	536	54	[14]
	AdkR	CCGTCAACTTTCGCGTATTT			
*blaTEM*	*TEM-F*	GCGGAACCCCTATTTG	964	50	[15]
	*TEM-R*	TCTAAAGTATATATGAGTAAACTTGGTCTGAC			
*blaSHV*	*SHV-F*	TTCGCCTGTGTATTATCTCCCTG	854	50	[16]
	*SHV-R*	TTAGCGTTGCCAGTGYTCG			
*blaCTX*	*CTX-F*	ATGTGCAGYACCAGTAARGTKATGGC	593	60	[17]
	*CTX-R*	TGGGTRAARTARGTSACCAGAAYCAGCGG			
hipO	CJF	ACTTCTTTATTGCTTGCTGC	323	59	[18]
	CJR	GCCACAACAAGTAAAGAAGC			
glyA	CCF	GTAAAACCAAAGCTTATCGTG	126		
	CCR	TCCAGCAATGTGTGCAATG			
23S rRNA	23S rRNA F	TATACCGGTAAGGAGTGCTGGAG	650		
	23S rRNA R	ATCAATTAACCTTCGAGCACCG			

### Simulation study with *Campylobacter* strains

In house reference strains of *C. jejuni* and *C. coli* used in this study were provided by the International Centre for Diarrhoeal Disease Research, Bangladesh (icddr,b). According to *Campylobacter* methodology UNI EN ISO 10272–1:2017, a loopful of each of the strains preserved was inoculated into Preston broth (Oxoid, UK, prepared by adding Modified Preston *Campylobacter* Selective Supplement, *Campylobacter* Growth Supplement, and Lysed horse blood according to the manufacturer’s instruction) and incubated at 37°C for 5 hours followed by 42°C for 48 hours under microaerophilic condition [4]. The incubated Preston broth was then inoculated onto mCCDA agar (Oxoid, UK; prepared by adding CCDA Selective Supplement (SR0155, Oxoid, UK) to *Campylobacter* Blood-Free Selective Agar Base (CM0739, Oxoid, UK, according to the manufacturer’s instructions) and blood agar(Oxoid Ltd, UK) incubated under microaerophilic conditions at 42°C for 48 hours. The presumptive growth of *Campylobacter* on mCCDA was examined by Gram’s staining. Finally, *C. jejuni* and *C. coli* were identified by PCR to detect the *hipO* and *glyA* gene and internal control to detect *Campylobacter* 23S RNA gene respectively. The primer sequences used to detect them are shown in (Table 1).

For the simulation study, poultry cecal material collected from chickens was dried and then sterilized by autoclaving at 121°C for 15 minutes. The sterility of the cecal content matrix was verified by inoculating it onto blood agar and by finding no bacterial growth after 48 hours of aerobic or microaerophilic incubation. Then, the sterile matrix was divided into six inoculum groups, referred to as A, B, C, D, E and F, and inoculated by 0.5 McFarland standard of specific bacterial cultures (Table 2). Here, In house reference strain of *Enterococcus fecalis* and *E.coli* ATCC25922 were used in this study as control. One loopful of inoculum from each of group was then inoculated into Preston broth, incubated 5 hours at 37⁰C followed by 42°C for 48 hours under microaerophilic condition. A loopful of enriched Preston broth culture from each group was then inoculated onto mCCDA and incubated at 42⁰C for 48 hours. The growth yielded from each of the inoculum groups on mCCDA was recorded. Three similar replication tests with the same samples were also performed in this simulation study.

**Table 2:** Inoculum groups in the simulation study with different organisms in sterile chicken cecal matrix and their growth on mCCDA after pre-enrichment in Preston broth.

Inoculum group	Contents in the inoculum matrix	*C. jejuni*	*C. coli*	*E. coli*	*E. faecalis*
A	CCM + *C. jejuni*	+++		–	–
B	CCM + *C. coli*		+++	–	–
C	CCM + *C. jejuni *+ *C. coli*	*+*	*+++*	*–*	*–*
D	CCM + *C. coli *+ *C. jejuni *+ ESBL *E. coli*	–	–	+++	–
E	CCM + *C. coli *+ *C. jejuni *+ *E. coli* ATCC25922	*+*	*+++*	*–*	*–*
F	CCM + *E. faecalis*	–	–	–	–

[CCM= chicken cecal matrix, +++ = highest isolation efficiency, ++ = moderate isolation efficiency, + = low isolation efficiency, – = nil].

### Minimum inhibitory concentration of tazobactam sodium to ESBL producing *E. coli*

A total of 10 randomly selected ESBL producing *E. coli* strains were used to assess the minimum inhibitory concentration of Tazobactam sodium using the broth micro dilution method in accordance with Clinical and Laboratory Standards Institute (CLSI) guidelines [19]. Cation adjusted Muller Hinton Broth (MHB) II (Oxoid, UK) and Tazobactam sodium (Sigma-Aldrich, Saint Louis, MO, USA) were used in broth microdilution. *E. coli* ATCC25922 was used as a negative control. MIC values were interpreted following CLSI guidelines [19].

### Determination of Tazobactam concentration in Preston broth and mCCDA

Preston broth was prepared with Tazobactam at different concentration: 1, 4, 16, 64, 128 mg/L and inoculated with *C. jejuni*, *C. coli,* and/or an ESBL *E. coli* strain with inoculum turbidity of 0.5 McFarland standard (equivalent to growth of 1–2 × 10^8^ CFU/mL). After incubation under microaerophilic condition at 42°C for 48 hours, a sub-sample from each tube was inoculated onto a mCCDA plate, incubated at 42°C for a further 48 hours under microaerophilic condition. The growth yielded after incubation were then evaluated. mCCDA was also prepared with Tazobactam at five different concentrations: 1, 4, 16, 64 and 128 mg/L, and inoculated separately with sub-samples of the same *C. jejuni, C. coli* and/or ESBL *E. coli* isolate at 0.5 McFarland standard. After incubation at 42°C for 48 hours microaerophilic, possible growth of *Campylobacter* was observed.

### Ethics statement

This study was approved [CVASU/Dir (R&E) EC/2020/165/2/1] by the Ethics Committee of Chattogram Veterinary and Animal Sciences University (CVASU), Bangladesh. No protected species were sampled. Chickens were humanely killed at a designated establishment by cervical dislocation, under animal welfare guidelines.

## Results

### Confirmation of contaminants on mCCDA

Whitish finely granular colonies were observed on mCCDA and confirmed as colonies of *E. coli* phenotypically on MacConkey and EMB agar and by PCR.

### ESBL gene screening of *E. coli* isolates

**Fig 1:**
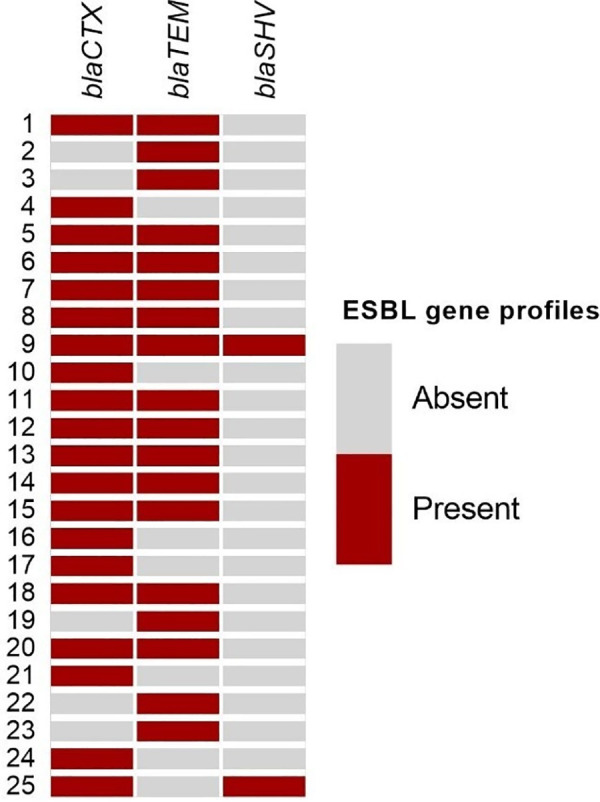
Distribution of ESBL genes in 25 *E. coli* strains isolated from growth on mCCDA during selective culture for *Campylobacter* from cecal contents of chickens.

All contaminant *E. coli* isolates were PCR positive for at least one of the tested ESBL genes (Fig 1). The highest frequency was observed for *bla*_CTX_ gene (n = 20, 80%) followed by 18 (72%) and 2 (8%) for *bla*_TEM_ and *bla*_SHV_ respectively.

### Verification of *C. jejuni* and *C. coli* strains for simulation study

**Fig 2:**
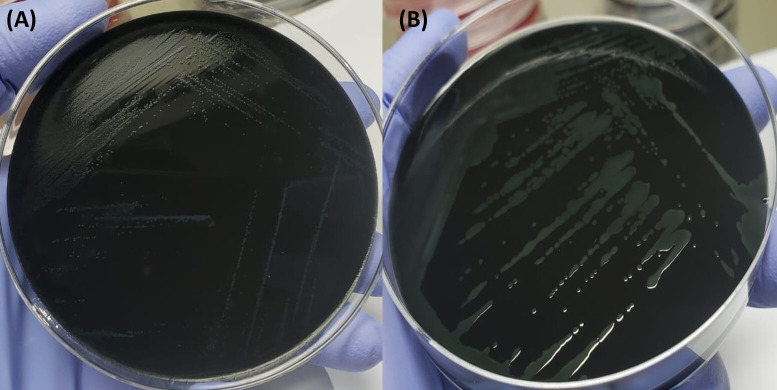
Depicting the growth of *C. jejuni* (A) and *C. coli* (B) on mCCDA.

**Fig 3:**
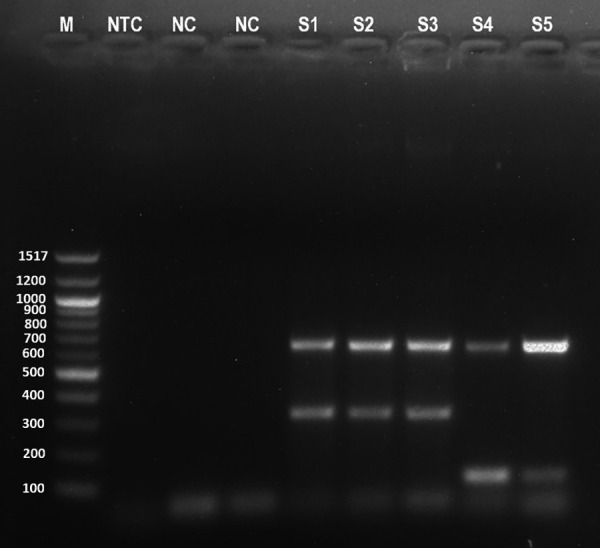
PCR products showing the specific amplicons for 23S rRNA (650 bp), hipO (323 bp), and glyA gene (126 bp) gene fragments targeting *Campylobacter* spp., *C. jejuni* and *C. coli* respectively. (M = 100 bp DNA ladder, NTC = No template control; NC = Negative control; S1 = *C. jejuni* from blood agar; S2 = *C. jejuni* from mCCDA; S3 = *C. jejuni* from Preston broth; S4 = *C. coli* from Blood agar; S5 = *C. coli* from mCCDA).

The *C. jejuni* strain collected from icddr,b produced small dry colonies on mCCDA while the *C. coli* strain produced comparatively larger, flat, watery and gray-colored colonies (Fig 2). PCR confirmed the presence of any *Campylobacter* spp., *C. jejuni* and *C. coli* as grown on different media (Fig 3).

### Simulation study

Simulation of selective culture for *Campylobacter* from chicken cecal contents in each replicate test in the presence of ESBL *E. coli* confirmed that only *E. coli* was detected on mCCDA (Table 2). Interspersed colonies characteristic of *C. jejuni* or *C. coli*, were not observed, confirming ESBL producing *E. coli* could mask the growth of *Campylobacter*. Neither *E. coli* ATCC25922 strain, an *E. coli* strain pan-susceptible to antimicrobials, nor *Enterococcus faecalis* were capable of growing on mCCDA after pre-enrichment through Preston broth. We found similar result in three replicate tests in this study. Colonies characteristic of *C. jejuni* and/or *C. coli* were seen when the cecal matrix inoculated with either/both were inoculated onto mCCDA after pre-enrichment using Preston broth.

### MIC of Tazobactam sodium

The MIC of Tazobactam for 10 ESBL *E. coli* strains found to host at least two ESBL genes was ≥ 128 mg/L for all (Table 3). Surprisingly, the MIC of Tazobactam to 7 of the 10 isolates was quite high, 128 mg/L, and for three other strains it was > 128.

**Table 3:** Tazobactam susceptibility of ESBL *E. coli* strains isolated from cecal swabs from live chickens with some identified ESBL genes.

*E. coli* Isolate no	ESBL gene	MIC (mg/L)*	Bird type	Sample source
1	*bla*_*CTX*_, *bla*_*TEM*_	>128	Deshi	Live bird market
2	*bla*_*CTX*_, *bla*_*TEM*_	>128	Sonali	Farm
3	*bla*_*CTX*_, *bla*_*TEM*_	128	Broiler	Live bird market
4	*bla*_*CTX*_, *bla*_*TEM*_, *bla*_*SHV*_	128	Deshi	Live bird market
5	*bla*_*CTX*_, *bla*_*TEM*_, *bla*_*SHV*_	128	Deshi	Live bird market
6	*bla*_*CTX*_, *bla*_*TEM*_	128	Broiler	Live bird market
7	*bla*_*CTX*_, *bla*_*TEM*_	128	Sonali	Farm
8	*bla*_*CTX*_, *bla*_*TEM*_	128	Broiler	Farm
9	*bla*_*CTX*_, *bla*_*TEM*_, *bla*_*SHV*_	>128	Sonali	Live bird market
10	*bla*_*CTX*_, *bla*_*TEM*_	128	Sonali	Live bird market

* minimum inhibitory concentration of tazobactam sodium.

### Verification of different concentrations of Tazobactam in Preston broth and in mCCDA

**Fig 4:**
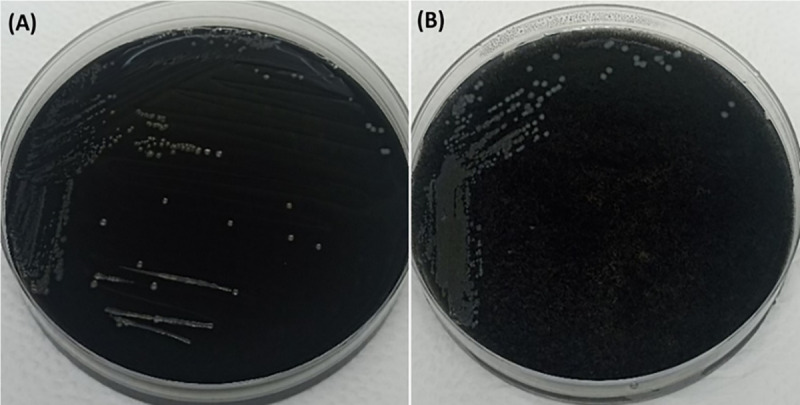
Growth of *Campylobacter* on mCCDA plate (A) *Campylobacter* growth following inoculation from Tazobactam supplemented-Preston broth at the level of 128 mg/L, inoculated with known *C. jejuni, C. coli* and an ESBL *E. coli* strain. **(B)** The growth of *Campylobacter* on mCCDA which was supplemented with Tazobactam at a concentration of 4 mg/L and inoculated directly with the same microorganisms without any pre-enrichment in Preston broth.

We assessed the addition of Tazobactam sodium into Preston broth by using six different concentrations (1, 4, 16, 64 and 128 mg/L) (Table 4). Although, growth of *E. coli* on mCCDA was seen for all the inoculums except 128 mg/L, no inhibitory effect of Tazobactam on *C. jejuni* or *C. coli* was observed. Interestingly, direct inoculation onto mCCDA supplemented with Tazobactam without enrichment found that ESBL *E. coli* failed to grow at concentrations of 4 mg/L or higher (Table 5, Fig 4). No inhibition of *Campylobacter* growth was detected following direct inoculation of mCCDA including Tazobactam concentrations up to 128 mg/L.

**Table 4:** Determination of the level of Tazobactam that could inhibit the growth of ESBL *E. coli* in Preston broth and recovering *C. coli,* and *C. jejuni* on mCCDA inoculated with inoculum from Preston broth pre-enriched with the three organisms.

Tazobactam concentration in Preston broth	Recovery from mCCDA without Tazobactam		
***C. coli***	***C. jejuni***	**ESBL *E. coli***
0mg/L	*+*	*+*	+++
1mg/L	+	+	+++
4mg/L	+	+	+++
16mg/L	+	+	+++
64mg/L	++	++	+++
128mg/L	+++	+++	+

[+++ = highest isolation efficiency, ++ = Moderate, + = Low, - = Nil].

**Table 5:** Determination of the tazobactam concentration added to mCCDA plates for direct recovery of *C. coli, and C. jejuni* without any pre-enrichment in Preston broth.

Tazobactam concentration in mCCDA	Recovery from mCCDA with Tazobactam		
	*C. coli*	*C. jejuni*	ESBL *E. coli*
**0mg/L**	***+***	***+***	**+++**
1mg/L	++	++	+++
4mg/L	+++	+++	–
16mg/L	+++	+++	*–*
64mg/L	+++	+++	*–*
128mg/L	+++	+++	*–*

[+++ = highest isolation efficiency, ++ = Moderate, + = Low, – = Nil].

Following confirmation of inhibition of ESBL *E. coli* but not *Campylobacter* by Tazobactam in mCCDA at 4 mg/L we deployed the revised protocol in *Campylobacter* surveillance from cecal samples collected from chickens. Based on the modified protocol applied, a total of 85 samples were investigated where 16 (18.8%) were found to be positive for *C. coli*, and 15 (17.65%) for *C. jejuni*, with no detection of ESBL *E. coli*.

## Discussion

The presence of *Campylobacter* in chicken feces poses a considerable risk of contamination to chicken meat leading to human infection. Effective isolation of *Campylobacter* for surveillance and diagnosis can be challenging due to overgrowth of non-*Campylobacter* contaminants on selective agar, e.g., ESBL producing *E. coli*. Supplementation of selective media using a beta lactamase inhibitor is one option to improve detection of *Campylobacter* from poultry cecal samples.

In this study, glossy white colonies were found to grow on mCCDA which obscured any *Campylobacter* present during selective culture of cecal content from chickens after pre-enrichment in Preston broth. These colonies were identified as ESBL producing *E. coli*, consistent with previous reports by others [8,9,11]. Cefoperazone, a selective substance in mCCDA media used to culture *Campylobacter*, contains a β-lactam ring that can be degraded by β-lactamase enzymes produced by beta lactamase producing *E.coli*, reducing the efficacy of selection [4,7,13]. ESBL producing *E. coli* grow faster than *Campylobacter*, including under microaerophilic circumstances [11], overgrowing and masking *Campylobacter* growth on mCCDA even after pre-enrichment culture in Preston broth. Among ESBL coding sequences *bla*_*CTX*_ was more frequently found than *bla*_*TEM*_ and *bla*_*SHV*_, reinforcing it as the most prevalent ESBL type in *E. coli* from poultry in all geographical areas [7,8,15,16,20–22]. The aberrant use of antibiotics in poultry, especially third-generation cephalosporins, could be linked to the acquisition and spread of ESBL genes in *E. coli* of poultry [23].

To test strategies to control ESBL *E. coli* during selective culture for *Campylobacter* reference *C. coli* and *C. jejuni* isolates were successfully recovered utilizing Preston broth and plating onto mCCDA in accordance with [4]. However, when mixed with an ESBL *E. coli* growth of *Campylobacter* was masked on mCCDA despite pre-enrichment in Preston broth, confirming the hypothesis that *Campylobacter* could not be recovered using this protocol when the original material, poultry cecal contents, is contaminated with ESBL *E. coli*. Contamination with *E. faecalis* did not prevent *Campylobacter* detection due to the inhibitory effects of cefoperazone in the mCCDA media [11]. Previous studies have suggested whenever there is a possibility of any ESBL producing organism(s) they need be nullified using a beta lactamase inhibitor like Tazobactum [11–13].

The breakpoint for an organism to be considered resistant to tazobactam is 4 mg/L [19]. The ESBL *E. coli* strains identified in this study as contaminants obscuring *Campylobacter* surveillance had MIC values of 128 mg/L to Tazobactam in broth culture. Such a high MIC in *E. coli* strains circulating in poultry could have been influenced by previous exposure to antimicrobials including ESBL inhibitors, but this was not known from the present study. Similarly high MICs have been reported for Tazobactam in *E. coli* from diverse sources in different geographical areas [24,25]. Culture of *C. coli* and *C. jejuni* in broth was unaffected by the presence of Tazobactam at this concentration. Culture on mCCDA revealed a lower MIC, with 4 mg/L inhibitory to ESBL *E. coli* but not *Campylobacter*, in line with previous findings that tazobactam up to 10 mg/L had no inhibitory impact on *Campylobacter* on mCCDA, while 1 mg/L suppressed ESBL producing *E. coli* [13]. In contrast, We found the concentration of Tazobactam as low as 128 mg/L that could be used in Preston broth to inhibit the growth ESBL producing *E. coli* strains circulating in commercial poultry in Bangladesh, enabling the isolation of *Campylobacter* later on mCCDA as part of sub-culturing selectively. The higher concentration of tazobactam in pre-enrichment broth was not corroborated with previous findings where 4 mg/L was enough to suppress ESBL *E.coli* in Bolton broth [26].

Here, the current study suggests that pre-enrichment in Preston broth can be removed from the existing ISO protocol and directly plating raw samples onto mCCDA supplemented with Tazobactam at a concentration of 4 mg/L would improve *Campylobacter* isolation efficiency along with the inhibition of ESBL producing *E.coli*. This modification offers a streamlined protocol compared to previous studies where classical pre-enrichment in Preston broth and selective enrichment on mCCDA were suggested [7,13,27–30]. When pre-enrichment is unavoidable due to very low *Campylobacter* occurrence, knowledge defining the Tazobactam MIC of the circulating *E. coli* strains can be used to refine the antibiotic concentration required to distinguish between the bacteria.

## Conclusion

Carriage of ESBL producing *E. coli* in poultry cecal contents poses a challenge to surveillance for *Campylobacter* using the ISO 10272:2017 protocol. The contrast between Tazobactam concentrations required to control ESBL *E. coli* in Preston broth compared to on mCCDA is an important consideration when using liquid or solid media for selective culture. Because Tazobactam is relatively expensive, mCCDA supplemented with Tazobactam at a concentration of 4 mg/L can be used as a direct inoculating solid media for effective isolation of *Campylobacter* in surveillance targeting poultry cecal content.

## Supporting information

S1 Table Raw results of ESBL producing gene detection. (DOCX)

S1 Fig Phenotypic observation of *E.coli* on mCCDA agar. (TIF)

S2 Fig Growth of *E.coli* observed on Mac conkey agar. (TIF)

S3 Fig Growth of *E.coli* observed on EMB agar. (TIF)

S4 Fig Growth of *E.coli* observed on Blood agar. (TIF)

S5 Fig Original image of PCR products showing the specific amplicons for 23S rRNA (650 bp), hipO (323 bp), and glyA gene (126 bp) gene fragments targeting *Campylobacter* spp., *C. jejuni* and *C. coli* respectively. (M = 100 bp DNA ladder, NTC = No template control; NC = Negative control; S1 = *C. jejuni* from blood agar; S2 = *C. jejuni* from mCCDA; S3 = *C. jejuni* from Preston broth; S4 = *C. coli* from Blood agar; S5 = *C. coli* from mCCDA).
(TIF)

## References

[1] Chon J-W, Hyeon J-Y, Choi I-S, Park C-K, Kim S-K, Heo S, et al. Comparison of three selective media and validation of the VIDAS Campylobacter assay for the detection of Campylobacter jejuni in ground beef and fresh-cut vegetables. J Food Prot. 2011;74(3):456–60. doi: 10.4315/0362-028X.JFP-10-302 21375884

[2] Ahmed R, León-Velarde CG, Odumeru JA. Evaluation of novel agars for the enumeration of Campylobacter spp. in poultry retail samples. J Microbiol Methods. 2012;88(2):304–10. doi: 10.1016/j.mimet.2011.12.011 22226753

[3] Kim J, Shin H, Park H, Jung H, Kim J, Cho S, et al. Microbiota analysis for the optimization of Campylobacter isolation from chicken carcasses using selective media. Frontiers in Microbiology. 2019;10:1381. 31293537 10.3389/fmicb.2019.01381 PMC6598470

[4] International Organization for Standardization. Microbiology of food and animal feeding stuffs – Horizontal method for detection and enumeration of Campylobacter spp. Part 1: Detection method. 2017.

[5] Koolman L, Whyte P, Bolton DJ. An investigation of broiler caecal Campylobacter counts at first and second thinning. J Appl Microbiol. 2014;117(3):876–81. doi: 10.1111/jam.12580 24946012

[6] Corry JE, Post DE, Colin P, Laisney MJ. Culture media for the isolation of campylobacters. Progress in Industrial Microbiology. 1995;34:129–62. 10.1016/0168-1605(95)00044-k 7662519

[7] Jasson V, Sampers I, Botteldoorn N, López-Gálvez F, Baert L, Denayer S, et al. Characterization of Escherichia coli from raw poultry in Belgium and impact on the detection of Campylobacter jejuni using Bolton broth. Int J Food Microbiol. 2009;135(3):248–53. doi: 10.1016/j.ijfoodmicro.2009.09.007 19786312

[8] Lina TT, Khajanchi BK, Azmi IJ, Islam MA, Mahmood B, Akter M, et al. Phenotypic and molecular characterization of extended-spectrum beta-lactamase-producing Escherichia coli in Bangladesh. PLoS One. 2014;9(10):e108735. doi: 10.1371/journal.pone.0108735 25302491 PMC4193765

[9] Moran L, Kelly C, Cormican M, McGettrick S, Madden RH. Restoring the selectivity of Bolton broth during enrichment for Campylobacter spp. from raw chicken. Letters in Applied Microbiology. 2011;52(6):614–8. doi: 10.1111/j.1472-765x.2011.03046.x 21488911

[10] Chon JW, Hyeon JY, Yim JH, Kim JH, Song KY, Seo KH. Improvement of modified charcoal-cefoperazone-deoxycholate agar by supplementation with a high concentration of polymyxin B for detection of Campylobacter jejuni and C. coli in chicken carcass rinses. Applied and Environmental Microbiology. 2012;78(5):1624–6. 22210208 10.1128/AEM.07180-11 PMC3294483

[11] Chon J-W, Kim H, Kim H-S, Seo K-H. Improvement of modified charcoal-cefoperazone-deoxycholate agar by addition of potassium clavulanate for detecting Campylobacter spp. in chicken carcass rinse. Int J Food Microbiol. 2013;165(1):7–10. doi: 10.1016/j.ijfoodmicro.2013.04.006 23685466

[12] Chon JW, Kim YJ, Kim HS, Kim DH, Kim H, Song KY, et al. Supplementation of Bolton broth with triclosan improves detection of Campylobacter jejuni and Campylobacter coli in chicken carcass rinse. International Journal of Food Microbiology. 2014;181:37–9. 24813626 10.1016/j.ijfoodmicro.2014.04.006

[13] Smith S, Meade J, McGill K, Gibbons J, Bolton D, Whyte P. Restoring the selectivity of modified charcoal cefoperazone deoxycholate agar for the isolation of Campylobacter species using tazobactam, a β-lactamase inhibitor. International Journal of Food Microbiology. 2015;210:131–5. 26119190 10.1016/j.ijfoodmicro.2015.06.014

[14] Wirth T, Falush D, Lan R, Colles F, Mensa P, Wieler LH, et al. Sex and virulence in Escherichia coli: an evolutionary perspective. Mol Microbiol. 2006;60(5):1136–51. doi: 10.1111/j.1365-2958.2006.05172.x 16689791 PMC1557465

[15] Olesen I, Hasman H, Aarestrup FM. Prevalence of beta-lactamases among ampicillin-resistant Escherichia coli and Salmonella isolated from food animals in Denmark. Microb Drug Resist. 2004;10(4):334–40. doi: 10.1089/mdr.2004.10.334 15650379

[16] Hasman H, Mevius D, Veldman K, Olesen I, Aarestrup FM. beta-Lactamases among extended-spectrum beta-lactamase (ESBL)-resistant Salmonella from poultry, poultry products and human patients in The Netherlands. J Antimicrob Chemother. 2005;56(1):115–21. doi: 10.1093/jac/dki190 15941775

[17] Miró E, Navarro F, Mirelis B, Sabaté M, Rivera A, Coll P, et al. Prevalence of clinical isolates of Escherichia coli producing inhibitor-resistant beta-lactamases at a University Hospital in Barcelona, Spain, over a 3-year period. Antimicrob Agents Chemother. 2002;46(12):3991–4. doi: 10.1128/AAC.46.12.3991-3994.2002 12435708 PMC132771

[18] Wang G, Clark CG, Taylor TM, Pucknell C, Barton C, Price L, et al. Colony multiplex PCR assay for identification and differentiation of Campylobacter jejuni, C. coli, C. lari, C. upsaliensis, and C. fetus subsp. fetus. J Clin Microbiol. 2002;40(12):4744–7. doi: 10.1128/JCM.40.12.4744-4747.2002 12454184 PMC154608

[19] CLSI. Performance Standards for Antimicrobial Disk and Dilution Susceptibility Tests for Bacteria Isolated From Animals. Wayne, PA: Clinical Laboratory Standards Institute; 2020. https://clsi.org/standards/products/veterinary-medicine/documents/vet01/

[20] Baez M, Espinosa I, Collaud A, Miranda I, Montano DD, Feria AL, et al. Genetic features of extended-spectrum β-lactamase-producing Escherichia coli from poultry in Mayabeque province, Cuba. Antibiotics. 2021;10(2):107. doi: 10.3390/antibiotics10020107 33499392 PMC7910960

[21] H 1 2, Mevius D, Veldman K, Olesen I, Aarestrup FM. β-lactamases among extended-spectrum β-lactamase (ESBL)-resistant Salmonella from poultry, poultry products and human patients in The Netherlands. Journal of Antimicrobial Chemotherapy. 2005;56(1):115–21. 15941775 10.1093/jac/dki190

[22] Rashid M, Rakib MM, Hasan B. Antimicrobial-resistant and ESBL-producing Escherichia coli in different ecological niches in Bangladesh. Infect Ecol Epidemiol. 2015;5:26712. doi: 10.3402/iee.v5.26712 26193990 PMC4507753

[23] Nahar S, Urmi UL, Ali T, Rumnaz A, Haque TA, Ara B, et al. ESBL Genes, blaTEM, blaOXA, and blaSHV in Poultry Gut Bacteria: An Endemic Public Health Burden in Bangladesh. Bangladesh Med Res Counc Bull. 2022;47(2):165–74. doi: 10.3329/bmrcb.v47i2.57775

[24] Farrell DJ, Flamm RK, Sader HS, Jones RN. Antimicrobial activity of ceftolozane-tazobactam tested against Enterobacteriaceae and Pseudomonas aeruginosa with various resistance patterns isolated in US hospitals (2011-2012). Antimicrobial Agents and Chemotherapy. 2013;57(12):6305–10. 24100499 10.1128/AAC.01802-13 PMC3837887

[25] López-Cerero L, Picón E, Morillo C, Hernández JR, Docobo F, Pachón J, et al. Comparative assessment of inoculum effects on the antimicrobial activity of amoxycillin-clavulanate and piperacillin-tazobactam with extended-spectrum beta-lactamase-producing and extended-spectrum beta-lactamase-non-producing Escherichia coli isolates. Clin Microbiol Infect. 2010;16(2):132–6. doi: 10.1111/j.1469-0691.2009.02893.x 19614715

[26] Chon J-W, Kim Y-J, Rashid F, Sung K, Khan S, Kim H, et al. Improvement of Bolton broth by supplementation with tazobactam for the isolation of Campylobacter from chicken rinses. Poult Sci. 2018;97(1):289–93. doi: 10.3382/ps/pex169 29112760

[27] Habib I, Uyttendaele M, De Zutter L. Evaluation of ISO 10272: 2006 standard versus alternative enrichment and plating combinations for enumeration and detection of Campylobacter in chicken meat. Food microbiology. 2011;28(6):1117–21. 21645809 10.1016/j.fm.2011.03.001

[28] Kiess AS, Parker HM, McDaniel CD. Evaluation of different selective media and culturing techniques for the quantification of Campylobacter ssp. from broiler litter. Poult Sci. 2010;89(8):1755–62. doi: 10.3382/ps.2009-00587 20634534

[29] Musgrove MT, Berrang ME, Byrd JA, Stern NJ, Cox NA. Detection of Campylobacter spp. in ceca and crops with and without enrichment. Poultry Science. 2001;80(6):825–8. 10.1093/ps/80.6.825 11441854

[30] Ugarte-Ruiz M, Gómez-Barrero S, Porrero MC, Alvarez J, García M, Comerón MC, et al. Evaluation of four protocols for the detection and isolation of thermophilic Campylobacter from different matrices. J Appl Microbiol. 2012;113(1):200–8. doi: 10.1111/j.1365-2672.2012.05323.x 22533742

