# Supplementary material for: Optimizing ISO standard microbiological techniques for isolating Campylobacter from poultry samples amidst challenges from extended spectrum beta lactamase producing Escherichia coli
Source: PLoS One. 2025 Jul 31;20(7):e0327963. doi: 10.1371/journal.pone.0327963 (PMC12313064; doi:10.1371/journal.pone.0327963)
Supplement: S1 Table — (DOCX) [file pone.0327963.s001.docx]

| Isolates no | bla CTX | bla TEM | blaSHV |
| --- | --- | --- | --- |
| 1 | 100 | 100 | 200 |
| 2 | 200 | 100 | 200 |
| 3 | 200 | 100 | 200 |
| 4 | 100 | 200 | 200 |
| 5 | 100 | 100 | 200 |
| 6 | 100 | 100 | 200 |
| 7 | 100 | 100 | 200 |
| 8 | 100 | 100 | 200 |
| 9 | 100 | 100 | 100 |
| 10 | 100 | 200 | 200 |
| 11 | 100 | 100 | 200 |
| 12 | 100 | 100 | 200 |
| 13 | 100 | 100 | 200 |
| 14 | 100 | 100 | 200 |
| 15 | 100 | 100 | 200 |
| 16 | 100 | 200 | 200 |
| 17 | 100 | 200 | 200 |
| 18 | 100 | 100 | 200 |
| 19 | 200 | 100 | 200 |
| 20 | 100 | 100 | 200 |
| 21 | 100 | 200 | 200 |
| 22 | 200 | 100 | 200 |
| 23 | 200 | 100 | 200 |
| 24 | 100 | 200 | 200 |
| 25 | 100 | 200 | 100 |

**S1 Table.** Raw results of ESBL producing gene detection

[Here 100=positive, 200= negative]
