# Supplementary figures and images for: Optimizing ISO standard microbiological techniques for isolating Campylobacter from poultry samples amidst challenges from extended spectrum beta lactamase producing Escherichia coli
Source: PLoS One. 2025 Jul 31;20(7):e0327963. doi: 10.1371/journal.pone.0327963 (PMC12313064; doi:10.1371/journal.pone.0327963)

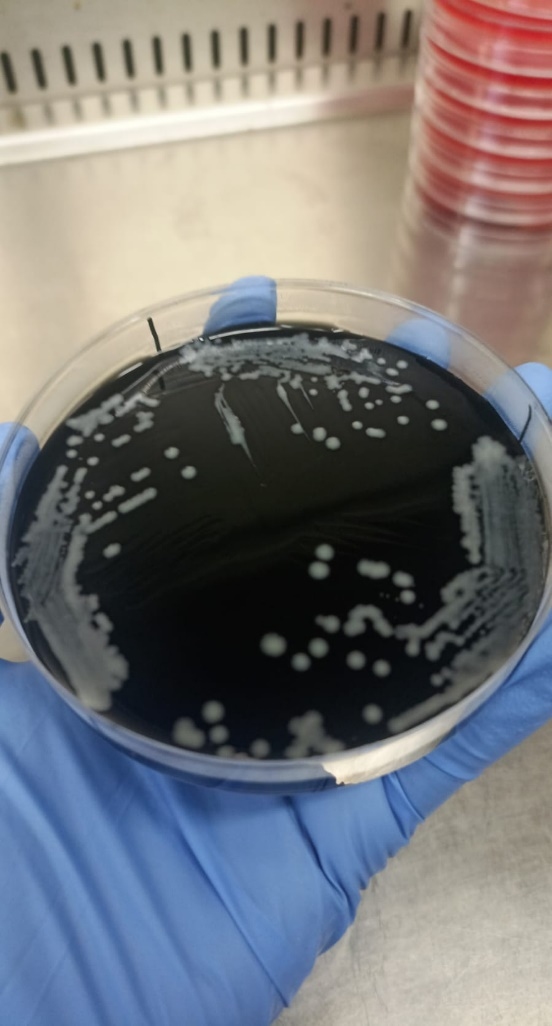

Supplement: S1 Fig — (TIF) [file pone.0327963.s002.tif]

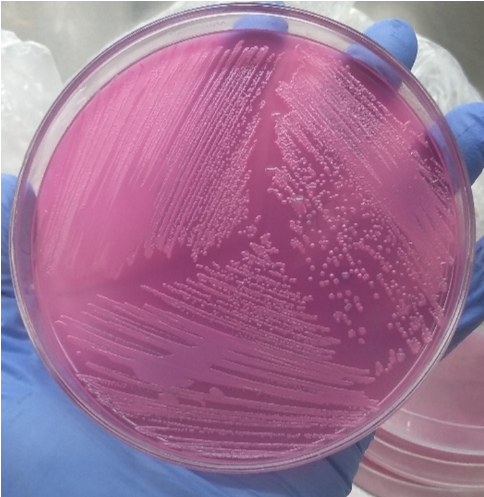

Supplement: S2 Fig — (TIF) [file pone.0327963.s003.tif]

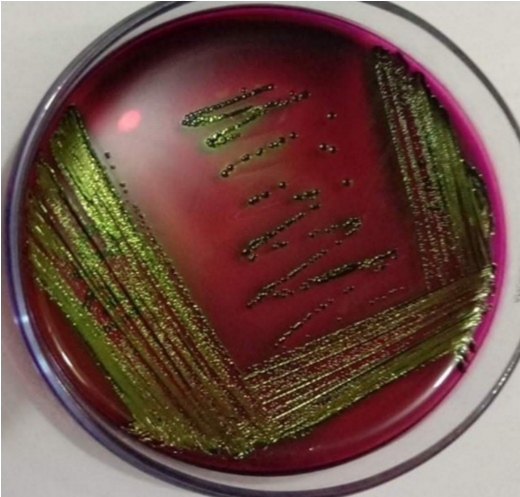

Supplement: S3 Fig — (TIF) [file pone.0327963.s004.tif]

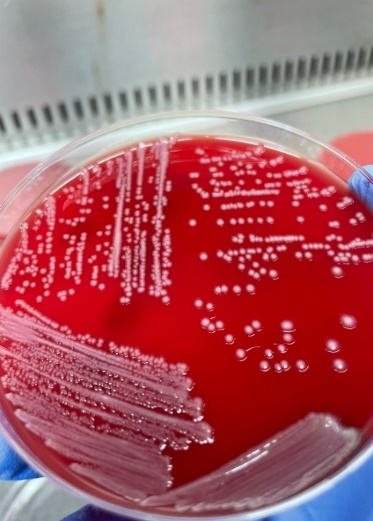

Supplement: S4 Fig — (TIF) [file pone.0327963.s005.tif]

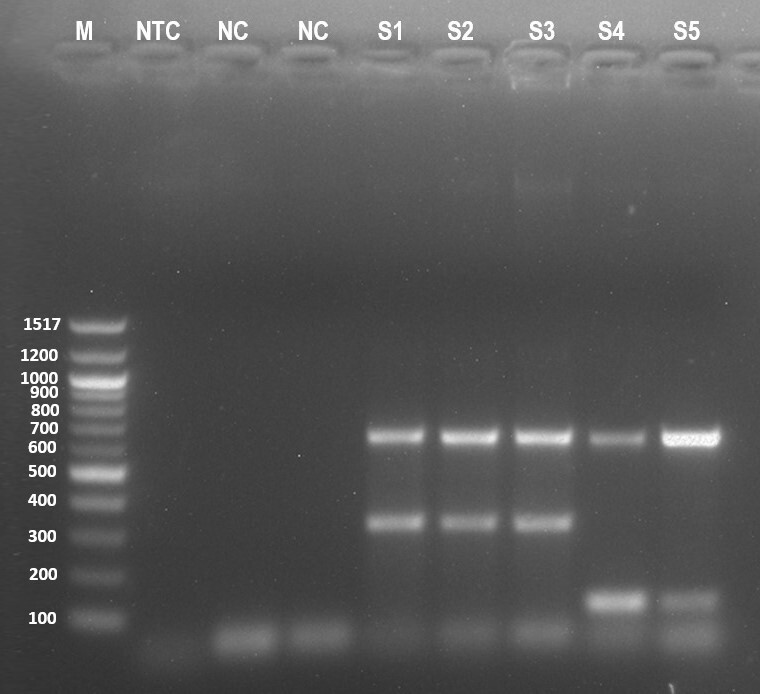

Supplement: S5 Fig — (M = 100 bp DNA ladder, NTC = No template control; NC = Negative control; S1 = C. jejuni from blood agar; S2 = C. jejuni from mCCDA; S3 = C. jejuni from Preston broth; S4 = C. coli from Blood agar; S5 = C. coli from mCCDA). (TIF) [file pone.0327963.s006.tif]
